# The Research on Borehole Stability in Depleted Reservoir and Caprock: Using the Geophysics Logging Data

**DOI:** 10.1155/2013/965754

**Published:** 2013-10-21

**Authors:** Junliang Yuan, Jingen Deng, Yong Luo, Shisheng Guo, Haishan Zhang, Qiang Tan, Kai Zhao, Lianbo Hu

**Affiliations:** ^1^State Key Laboratory of Petroleum Resources and Prospecting, China University of Petroleum, Beijing 102249, China; ^2^CNOOC China Limited, Shanghai Branch, Shanghai 200030, China; ^3^College of Petroleum Engineering in Xi'an Shiyou University, Xi'an 710065, China

## Abstract

Long-term oil and gas exploitation in reservoir will lead to pore pressure depletion. The pore pressure depletion will result in changes of horizontal in-situ stresses both in reservoirs and caprock formations. Using the geophysics logging data, the magnitude and orientation changes of horizontal stresses in caprock and reservoir are studied. Furthermore, the borehole stability can be affected by in-situ stresses changes. To address this issue, the dehydration from caprock to reservoir and roof effect of caprock are performed. Based on that, the influence scope and magnitude of horizontal stresses reduction in caprock above the depleted reservoirs are estimated. The effects of development on borehole stability in both reservoir and caprock are studied step by step with the above geomechanical model.

## 1. Introduction

During the development of oil and gas fields, the pore pressure in reservoir will decease dramatically due to the hydrocarbon exploitation. Decades of development experience reveal that the pore pressure change has great impact on horizontal in-situ stresses. In some cases, the significant change of in-situ stresses may even activate the instable faults. The accurate in-situ stresses evaluation is one of the most important factors in safe drilling and hydraulic fracturing design [1–3]. For these reasons, researchers have done extensive works on the changes of in-situ stresses caused by oil development [4–6].

Previous studies had paid much attention to the reservoir formations and the magnitude change of in-situ stresses ignoring the orientation change and the change in caprock. However, some leak off tests in oil fields indicate that the in-situ stresses in the caprock may also change significantly due to the depletion of reservoir pore pressure. So, it is meaningful and imperative to study the in-situ stress change in caprock.

## 2. Geomechanical Effect of Exploitation on In-Situ Stress Magnitude

The magnitude change of in-situ stresses in reservoir caused by oil development has been investigated by several authors. Addis [7] showed that the magnitude of the change of the minimum horizontal stress has a linear relationship with that of the pore pressure by analyzing the in-situ testing data of oil and gas fields of North America, North Sea such as Magnus, West Sole, and Wytch Farm fields. According to Addis, the minimum horizontal stress will decrease as the pore pressure decreases. But for different fault block fields, the proportional coefficient is generally different. For reservoirs with different boundary conditions and properties, Amadei et al. [8] provided the analytic solution of the proportional coefficient *K* with uniaxial compression model. The analytic solution is presented in Table 1.

According to the porous linear elastic theory, supposing the reservoir is homogeneous, the change value of in-situ stresses (Δ*σh*) has a linear relationship with that of the pore pressure (Δ*Pp*) under the uniaxial compression condition. So, the horizontal in-situ stresses of formations developed over a long period of time can be calculated by ((1a), (1b), and (1c)). All the other parameters can be obtained by geophysical logging data, for example, *V*_*p*_ and *V*_*s*_ ((1b)–(1c)) [9–12]:

(1a) $$\begin{array}{c} \sigma_{H}^{\prime }=\;\sigma_{H}-K\cdot \alpha \cdot {\Delta P}_{p}\\ \sigma_{h}^{\prime }=\;\sigma_{h}-K\cdot \alpha \cdot {\Delta P}_{p}, \end{array}$$

(1b) $$\begin{array}{c} \sigma_{H}=\left(\frac{\nu }{1-\nu }+A\right)\left(\sigma_{V}-{\alpha P}_{p}\right)+{\alpha P}_{p}\\ \sigma_{h}=\left(\frac{\nu }{1-\nu }+B\right)\left(\sigma_{V}-{\alpha P}_{p}\right)+{\alpha P}_{p},\\ E_{d}=\frac{\rho V_{s}^{2}\left(3V_{p}^{2}-4V_{s}^{2}\right)}{\left(V_{p}^{2}-2V_{s}^{2}\right)}, \end{array}$$

(1c) $$\begin{array}{c} \nu_{d}=\frac{\left(V_{p}^{2}-2V_{s}^{2}\right)}{2\left(V_{p}^{2}-2V_{s}^{2}\right)}\\ E_{d}=\frac{\rho V_{s}^{2}\left(3V_{p}^{2}-4V_{s}^{2}\right)}{\left(V_{p}^{2}-2V_{s}^{2}\right)}, \end{array}$$

where *σH*′ and *σh*′ are the present maximum and minimum horizontal principle stresses, respectively; *σH* and *σh* are the original values; *K* is the proportional coefficient; *α* is the effective stress coefficient, Δ*Pp* is the change of the pore pressure; *E*, *v* are the elastic modulus and Poisson's ratio, respectively, which can be calculated by the *E*_*d*_ and *v*_*d*_ in (1c). Φ is the internal frictional angle of fault, *A*, *B* are the coefficients of tectonic stress, *V*_*p*_ is compressive wave velocity, m/s, and Vs is shear wave velocity, m/s.

Morita et al. [13] show that if the ratio of reservoir thickness and radius is smaller than 0.1 and the ratio between shear modulus of reservoir and caprock (GR/GC) is between 0.2 and 1.5, the result of (1a), (1b), and (1c) is relatively accurate.

## 3. Geomechanical Effect of Exploitation on In-Situ Stress Orientation

**Figure 1:**
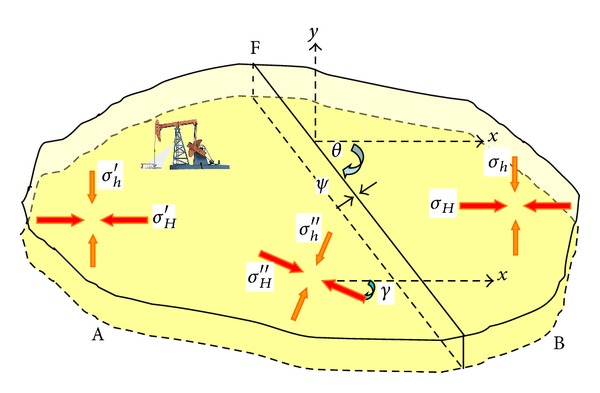
Schematic diagram of geomechanical model.

For depleted fault block reservoirs, if the dip of fault and the orientation of horizontal stresses are not parallel, shear stress will present near the fault. Thus, the orientation of in-situ stresses near the fault is not the same as that of the in-situ stress far away from the fault. Sonder [14] analyzed the effect of development with geomechanical model (Figure 1). The assumptions of the model include (1) the fault F is impervious; (2) the change of formation temperature can be neglected; (3) the formation is homogeneous.

It can be seen from Figure 1 that the original orientation of horizontal stresses is parallel to the *x*-axis and the fault F is at an angle of *θ* to the orientation of maximum horizontal stress. The fault F divides the formation into two blocks, A and B. The pore pressure in the area A decreases dramatically due to the long-period development, while the pore pressure of area B maintains the original value. The pore pressure difference between area A and B generates the normal traction force *ψ* at both sides of the fault [15]. Because the direction of force *ψ* and the *x*-axis are not parallel, the orientation of horizontal in-situ stresses will rotate at some angle *γ*, and the angle *γ* can be calculated by the following equations [14]:

(2) $$\begin{array}{c} \gamma =\frac{1}{2}\tan^{-1}\left[\frac{K\cdot \partial \cdot q\sin2\theta }{1+K\cdot \partial \cdot q\cos2\theta }\right],\\ q=\frac{{-\Delta P}_{p}}{\sigma_{H}-\sigma_{h}}, \end{array}$$

where *γ* is the deflected angle of the horizontal stress near the fault; *K* is the scaling factor; *α* is the effective stress coefficient; Δ*Pp* is the change of the pore pressure; *θ* is the angel between the regional horizontal maximum stress and the dip of the fault.

**Figure 2:**
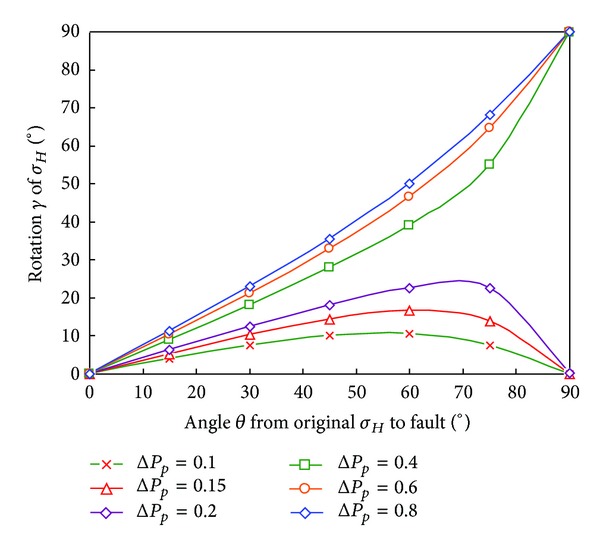
The relationship between deflected angle and the angle *θ*.

According to the previous equations, the value of parameters can be assumed as follows: Poisson's ratio *υ* = 0.22; Biot's coefficient *α* = 0.8; the maximum horizontal in-situ stress *σ*_*H*_ = 1.8 g/cm^3^; the minimum horizontal in-situ stress *σ*_*h*_ = 1.6 g/cm^3^, and the depletion of pore pressure ranges from 0.1 g/cm^3^ to 0.8 g/cm^3^, and then the relationship of the deflected angle *γ* and the angle *θ* is illustrated in Figure 2.

## 4. Geomechanical Effect of Exploitation on Caprock In-Situ Stresses

For severely depleted reservoir, the in-situ stresses in caprock will change due to the draining effect and top plate effect [16–18]. Morita and Fuh [19] showed that the change of the in-situ stresses in caprock cannot be ignored after modeling its change degree with finite element model.

The pore pressure difference between the reservoir and caprock will drive the fluid from caprock into reservoir. Though the permeability of caprock is very low, decades of seepage will affect the reservoir in-situ stresses [19–23]. This process is called draining effect of caprock. What's more, the decrease of pore pressure causes the increasing of matrix stress and the caprock will deform accordingly. This phenomenon is called top plate effect. The influence of pore pressure depletion in the caprock can be calculated by the following equations [19]:

*Initial Conditions.* Consider

(3) $$\begin{array}{c} P=P_{o}\;\text{For}\;0<z<\infty ,\;t=0,\\ {\text{P}\;|\;}_{z=0}=P_{o}-\left(P_{o}-P_{r}\right)\frac{t}{t_{c}}\;\text{for}\;z=0,\;t>0. \end{array}$$

*Governing Equations.* Consider

(4) $$\begin{array}{c} \frac{\partial^{2}P}{{\partial z}^{2}}=\frac{\phi \mu c}{C_{1}k}\frac{\partial P}{\partial t},\\ {E\Delta \varepsilon }_{v}={\Delta \sigma }_{v}-\upsilon \left({\Delta \sigma }_{H}+{\Delta \sigma }_{h}\right)-\left(1-2\upsilon \right)\Delta P,\\ {E\Delta \varepsilon }_{v}={\Delta \sigma }_{v}-\upsilon \left({\Delta \sigma }_{H}+{\Delta \sigma }_{h}\right)-\left(1-2\upsilon \right)\Delta P,\\ {E\Delta \varepsilon }_{h}={\Delta \sigma }_{h}-\upsilon \left({\Delta \sigma }_{v}+{\Delta \sigma }_{H}\right)-\left(1-2\upsilon \right)\Delta P. \end{array}$$

*Boundary Conditions.* Consider

(5) $$\begin{array}{c} {\Delta \varepsilon }_{H}={\Delta \varepsilon }_{h},\\ {\Delta \sigma }_{v}=0. \end{array}$$

Based on the above equations, at time *t*_*c*_, the pore pressure with respect to *z* can be given by the following equations [19]:

(6) $$\begin{array}{c} P_{p}=P_{o}-\left(P_{o}-P_{r}\right)\cdot \zeta , \end{array}$$

(7) $$\begin{array}{c} \zeta =\left(1+\frac{z^{2}}{{2\lambda t}_{c}}\right)\mathrm{erf}c\frac{z}{2\sqrt{{\lambda t}_{c}}}-\frac{z}{\sqrt{\pi {\lambda t}_{c}}}\exp\left(\frac{{-z}^{2}}{{4\lambda t}_{c}}\right), \end{array}$$

where *λ* = *c*_1_ *k*/*ϕμc*, *Pp* is the present pore pressure in the caprock formations; *P*_*o*_ is the original pore pressure of the caprock; *P*_*r*_ is the present pressure of the depleted reservoir; *z* is the vertical distance from the top of the reservoir to the interest point in the caprock; *k*, *φ*, *μ* are the permeability, porosity, and fluid viscosity of the caprock, respectively. *C*_1_ is the compressibility of the fluid; *c*_1_ is the unit transformation ratio 2.64 × 10^−4^; *t*_*C*_ is the development time, years.

**Figure 3:**
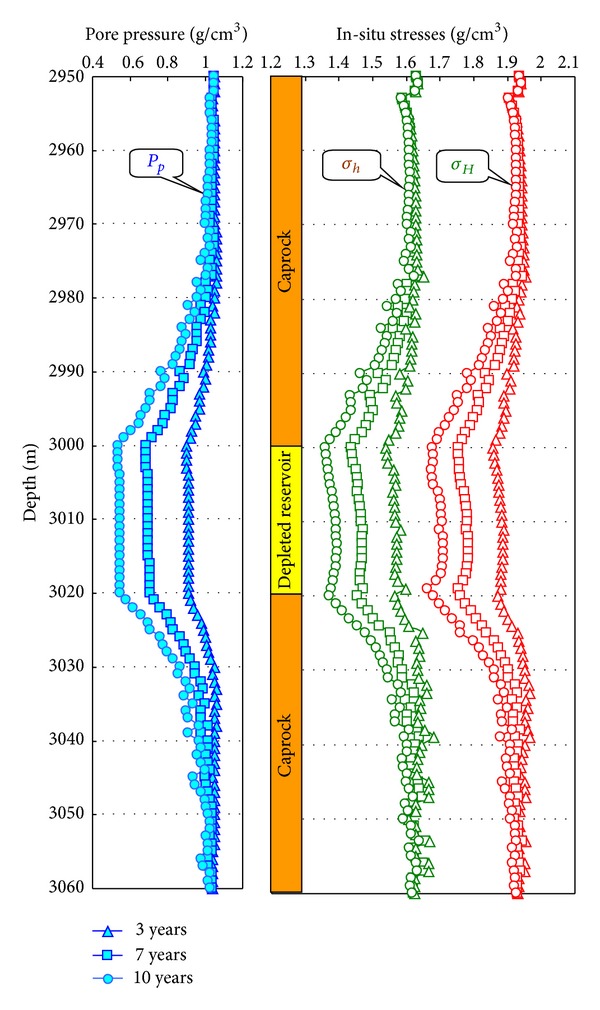
Influence of development on the in-situ stresses of caprock and reservoir with different development times.

According to (3)–(7), we can calculate the pore pressure of caprock at different depth, by substituting the results into (1a), (1b), and (1c), the in-situ stress of the caprock after the depletion of the reservoir is gained. The change of caprock formation of a certain field in BoHai Gulf Basin is present in Figure 3 based on the geophysics logging data. The involved parameters have the same value in Section 3.

In Figure 3, the filled triangular, square, and circle symbols represent pore pressure when the development time of reservoir is 3 years, 7 years, and 10 years, respectively. The open green triangular, square, and circle symbols represent minimum horizontal stresses when the development time of reservoir is 3 years, 7 years, and 10, years respectively. The open red triangular, square, and circle symbols represent maximum horizontal stresses when the development time of reservoir is 3 years, 7 years, and 10 years, respectively.

## 5. Borehole Stability Analysis

**Figure 4:**
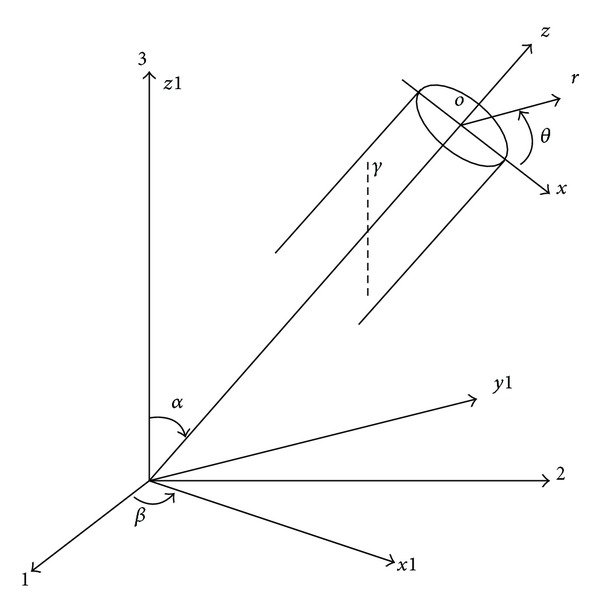
Coordinate conversion.

The in-situ stress calculated by former chapter should be transformed form the geodetic coordinate systems (1, 2, 3) to borehole coordinate system (*x*, *y*, *z*). The coordinate conversion is presented in Figure 4. The conversion relation is as follows:

(8) $$\begin{array}{c} \left[\begin{array}{ccc} \sigma_{xx} & \sigma_{xy} & \sigma_{xz}\\ \sigma_{yx} & \sigma_{yy} & \sigma_{yz}\\ \sigma_{zx} & \sigma_{zy} & \sigma_{zz} \end{array}\right]=\left[L\right]\left[\begin{array}{ccc} \sigma_{H} & 0 & 0\\ 0 & \sigma_{h} & 0\\ 0 & 0 & \sigma_{V} \end{array}\right]{\left[L\right]}^{T}, \end{array}$$

where *L* is the coordinate system transformation matrix. Consider

(9) $$\begin{array}{c} \left[L\right]=\left[\begin{array}{ccc} \cos\beta \cos\alpha & \cos\beta \sin\alpha & -\sin\beta \\ -\sin\alpha & \cos\alpha & 0\\ \sin\beta \cos\alpha & \sin\beta \sin\alpha & \cos\beta \end{array}\right]. \end{array}$$

The basic approach to solve such problem consists of the stress distribution around wellbore and failure criterion, and calculating the safe mud-weight windows subsequently. Based on reliable research [9, 23–27], the modeling is as follows.

The stress states of near wellbore are, respectively,

(10) σr=R2r2Pi+(σxx+σyy)2(1−R2r2) +(σxx+σyy)2(1+3R4r4−4R2r2)cos⁡⁡2θ +σxy(1+3R4r4−4R2r2)sin2θ +δ[α(1−2ν)2(1−ν)(1−R2r2)−ϕ](Pi−Pp),

(11) σθ=−R2r2Pi+(σxx+σyy)2(1+R2r2) −(σxx−σyy)2(1+3R4r4)cos⁡⁡2θ −σxy(1+3R4r4−4R2r2)sin2θ +δ[α(1−2ν)2(1−ν)(1−R2r2)−ϕ](Pi−Pp),

(12) σz=σzz−ν[2(σxx−σyy)(Rr)2×cos⁡⁡2θ+4σxy(Rr)2sin⁡2θ] +δ[α(1−2ν)1−ν−ϕ](Pi−Pp),

(13) σrθ=σxy(1−3R4r4+2R2r2)cos⁡⁡2θ,

The principle stresses on borehole wall are calculated as follows [28, 29]:

(14) σ1=σz+σθ2+[σz−σθ2]2+σθz2,

(15) σ2=σr=Pm−δf(Pm−Pp),

(16) σ3=σz+σθ2−[σz−σθ2]2+σθz2.

The rock mechanical parameters, for example, uniaxial compressive strength, can be obtained by [30–32]

(17) $$\begin{array}{c} \sigma_{c}=\left(0.0045+{0.0035V}_{\mathrm{cl}}\right)E_{d}, \end{array}$$

(18) $$\begin{array}{c} V_{\mathrm{cl}}=\frac{2^{\text{GCUR}\cdot I_{\text{GR}}}-1}{2^{\text{GCUR}}-1}, \end{array}$$

(19) $$\begin{array}{c} I_{\text{GR}}=\frac{\text{GR}-{\text{GR}}_{\min}}{{\text{GR}}_{\max}-{\text{GR}}_{\min}}, \end{array}$$

(20) $$\begin{array}{c} S_{t}=\frac{\sigma_{c}}{12}, \end{array}$$

where *V*_cl⁡_ is shale content, GR is gamma ray log, GR_min⁡_ and GR_max⁡_ are, respectively, the gamma of pure sand and pure shale; G_GCUR_ is Hilchie index, which is related to geologic period, and it could be 3.7 for Tertiary and 2 for older formation; *I*_GR_ is shale content index.

The collapse pressure (*P*_*c*_) and fracture pressure (*P*_*f*_) can be calculated with (21) and (22), respectively,

(21) $$\begin{array}{c} \left(\sigma_{1}-\alpha \cdot P_{p}\right)\geq \left(\sigma_{3}-\alpha \cdot P_{p}\right)\cdot \tan^{2}\left(\frac{\pi }{4}+\frac{\phi }{2}\right)+\sigma_{c}, \end{array}$$

(22) $$\begin{array}{c} \sigma_{3}-\alpha \cdot P_{p}\leq -S_{t}, \end{array}$$

where  *σ*_3_ is the minimum stress on the borehole wall, *P*_*p*_ is critical pore pressure, and *S*_*t*_ is tensile strength.

**Figure 5:**
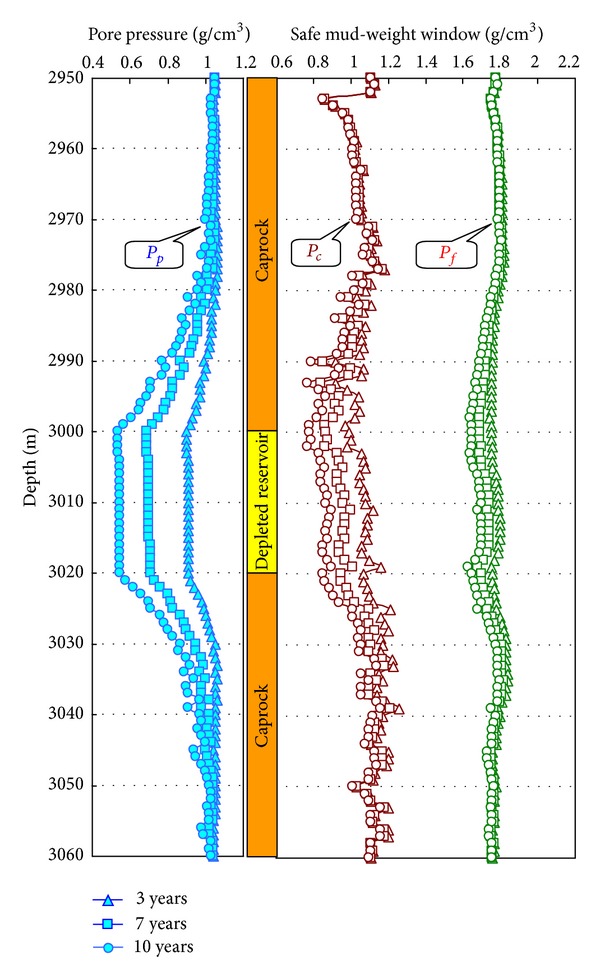
The safe mud-weight window of caprock formation and depleted reservoir with different development times.

Based on the above equations and geomechanical parameters (shown in Figure 4), the safe mud-weight window of depleted reservoir and caprock formations can be obtained. The calculation results of the vertical well are shown in Figure 5. The results show that the collapse pressure and fracture pressure both reduce with the development time.

**Figure 6:**
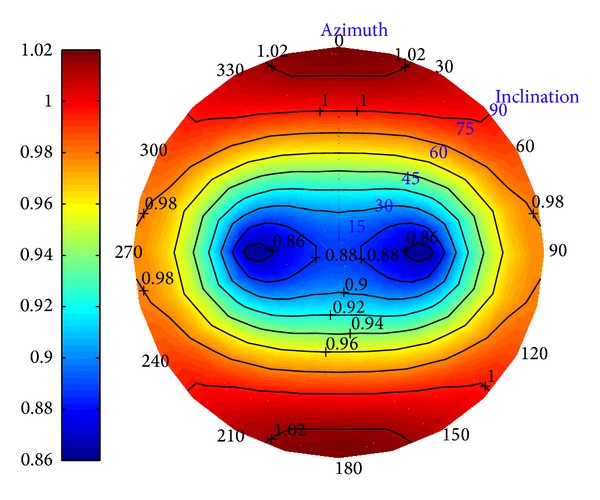
Collapse pressure versus well azimuth in the depleted reservoir with *t*_*c*_ = 7 years.

**Figure 7:**
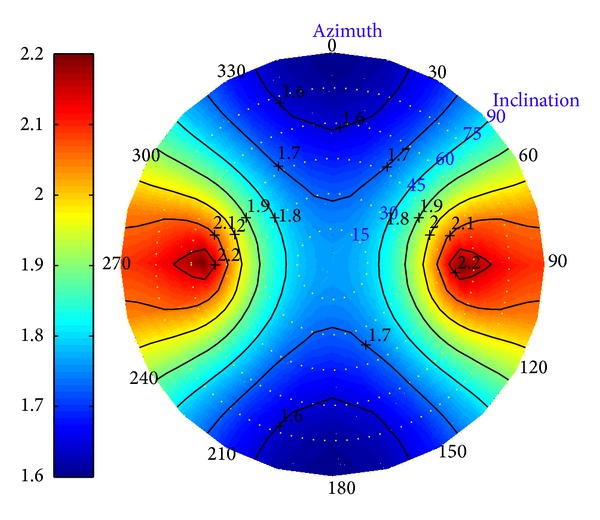
Fracture pressure versus well azimuth in the depleted reservoir with *t*_*c*_ = 7 years.

Figures 6 and 7 show the current collapse pressure and fracture pressure versus the borehole inclination and azimuth in depleted reservoir after 7 years of development. They illustrate that the variation of critical mud-weight is apparent, which are the safe ranges of drilling mud density to avoid borehole fracturing.

## 6. Conclusions

(1) The field development has a great effect on both magnitude and orientation of in-situ stresses in reservoir, and the influence degree depends on the rock mechanical properties of reservoir, sealing of the fault, and the magnitude of original horizontal in-situ stresses.

(2) The oil and gas field development will also have a significant impact on the in-situ stresses in caprock. The impact is related to the development time, fluid viscosity, and rock permeability. Long-term development may affect the in-situ stresses of caprock in dozens meters above the reservoir.

(3) The geomechanics effect of exploitation on in-situ stress could reduce the collapse pressure and the fracture pressure significantly in reservoir, and the effection can not be negligible in caprock formation near the reservoir.

## Figures and Tables

**Table 1:** The value of proportional coefficient *K*.

Formation property	Boundary conditions		
	No fault	Fault boundary	
		Normal fault	Thrust fault
Isotropic formation	$1-\frac{\nu }{1-\nu }$	(1) $\frac{2\sin\phi }{1+\sin\phi }$	1 − *ν*(*K*_*p*_ + 1)
		(2) $\frac{\sin\phi +1-2\nu }{1+\sin\phi }$	

Anisotropic formation	$1-\frac{\nu^{\prime }E}{E^{\prime }(1-\nu )}$	(1) $\frac{2\sin\phi }{1+\sin\phi }$	$\left[1-K_{p}\nu -\frac{\nu^{\prime }E}{E^{\prime }}\right]$
		(2) $\left[1-\frac{\nu }{K_{p}}-\frac{\nu^{\prime }E}{E^{\prime }}\right]$	
